# Comparison of Blood and Tissue Eosinophil Count and Blood IgE in Patients with Chronic Sinusitis and Nasal Polyps

**DOI:** 10.1155/2021/6680676

**Published:** 2021-02-10

**Authors:** Masoud Asghari, Shadi Izadpanahi, Mina Heidari Esfahani

**Affiliations:** ^1^Department of Otorhinolaryngology, School of Medicine, Birjand University of Medical Sciences, Iran; ^2^Birjand University of Medical Sciences, Iran; ^3^Shiraz University of Medical Science (SUMS), Iran

## Abstract

**Background:**

The inflammatory mucosa of the sinus cavities is called sinusitis and is divided into various types based on its appearance and sign. Chronic rhinosinusitis is an inflammatory-infectious disease that involves the frontal, sphenoid, ethmoid, and maxillary sinuses. Chronic sinusitis is a multifactorial disease and the range of causes varies from environmental factors to genetic factors. The purpose of this study was to compare blood and tissue eosinophils and serum IgE levels in patients with chronic sinusitis with nasal polyp in Vali-e-Asr hospital in 1397.

**Methods:**

In this descriptive-analytical study, the population under study included those with chronic sinusitis referred to Birjand Valiasr Hospital in 1397.3 cc of blood samples were taken 1 day before surgery to evaluate eosinophil counts and serum IgE levels. Also, samples taken from patients during surgery were counted, and then, 100 cells were counted, and eosinophil counts and percentages were calculated. The data were entered into the SPSS software after data collection.

**Results:**

This study was performed on 70 patients with chronic rhinosinusitis which included 43 men (61.4%) and 27 women (38.6%) with mean age of 39.11 ± 13 13.72 years. There was no significant difference between sex of patients and mean serum IgE level (*P* < 0.05). The mean percentage of eosinophils in blood samples and tissues of patients with chronic sinusitis was significantly increased with the increase in CT scan (*P* < 0.05).

**Conclusions:**

Tissue or blood eosinophilia was not observed in patients with chronic rhinosinusitis. Also, the mean eosinophil percentage of blood and tissue increased significantly in patients with increased scanning computed tomography (*P* < 0.05).

## 1. Introduction

Rhinosinusitis or sinusitis is an inflammation in the lining of the sinuses cavities. Untreated acute sinusitis becomes subacute and results in complete nasal congestion and obstruction. Incomplete treatment of this disease also results in chronic sinusitis. Severe histological changes occur in the mucosa of sinuses. These changes are somewhat irreversible [1]. Chronic rhinosinusitis is an inflammatory-infectious disease influencing frontal, sphenoid, ethmoid, and maxillary sinuses. If the disease lasts for more than 3 months, it will be called chronic sinusitis [2, 3]. The prevalence of this disease varies in different countries. It is estimated to be between 14 and 15%. It is one of the most common chronic diseases around the world [4, 5]. The prevalence of this disease in the world is 135 thousand people in whole population (13.5%). Some studies suggest that about 31 million new cases of sinusitis are diagnosed in the United States annually [6]. This disease is associated with complications. For this reason, it is considered as one of the 10 disabling diseases for patients [7]. Its symptoms include feeling of pressure and fullness in face, dryness, dry coughs, nasal congestion and obstruction, anterior and posterior nasal discharge, intermittent headaches, swelling and forehead or cheek erythema, olfactory dysfunction, edema, fatigue, and congestion of nasal mucosa [8–10]. This disease imposes high financial burden on Iran's health system. Some of the financial burden of this disease include the need for frequent outpatient visits, high number of diagnostic and therapeutic measures, and reduced productivity at work [11].

Mucosal inflammation caused by bacteria or fungi, environmental factors such as dry air, tobacco smoke, air pollution, socio-economic factors, history of asthma, and so on are among the factors affecting chronic rhinosinusitis with nasal and sinus polyposis [12]. Some of the disease complications include orbital cellulitis, periosteal abscess, osteomyelitis, intracerebral complications, and cavernous sinus thrombosis [2]. In this disease, the quality of life of patients is affected so that it can be compared with other chronic debilitating diseases [13]. Polyps are easily available for immunological and histological studies. For this reason, many studies and articles have been published in recent years about it. Nasal polyps have long been associated with rhinitis and asthma, but there is disagreement about the role of allergies in the etiology and pathogenesis of nasal polyps. Nasal polyps are characterized by the presence of lymphocytes in the tissue and the presence of neutrophils in patients' discharges [14]. Chronic rhinosinusitis manifests itself in the form of eosinophil infiltration in the nasal and sinus mucosa. Eosinophils are seen in patients with and without polyps. Inflammation of the nasal cavity and chronic sinusitis are directly associated with level of eosinophils in nasal discharges [15]. Also, a significant relationship has been reported between the level of eosinophils and nasal discharges in allergic patients [16]. Granular proteins of eosinophils are specifically toxic to the respiratory tract epithelium and are found in chronic areas of the epithelium in chronic rhinosinusitis. Given what was stated, it can be stated that eosinophils may play a major role in the physiopathology of chronic rhinosinusitis [17]. The aim of present study is to measure nasal eosinophil polyps and serum IgE levels and to compare the clinical symptoms and CT scan score of these patients in a specific time period with the percentage of eosinophilia and the effect of eosinophilia and IgE on the severity of the disease.

## 2. Materials and Methods

The statistical population of this descriptive-analytical study included all patients with chronic sinusitis with nasal polyps, referred to the ENT clinic of Valiasr Hospital in Birjand from 2018/3/21 to 2019/3/20. Inclusion criteria of the study included an age over 17 years and obtaining informed consent from subjects. Exclusion criteria included any renal and vascular disorders, malignancy, chemotherapy, and radiation therapy in the studied patients. Patients with clinical signs of chronic sinusitis were examined by an ENT specialist and without considering the patients' gender. After clinical and paraclinical tests, patients with a diagnosis of chronic sinusitis were finally selected. One day before surgery, 3 cc of blood samples was taken from patients to assess the number of eosinophils and serum IgE levels. Also, samples were taken from patients during the surgery, and they were stained. Then, 100 cells were counted, and the number and percentage of eosinophils were calculated. Patients' indications for surgery included the following cases: continued clinical symptoms or signs of chronic sinusitis including discharge from the nose and throat, headache and pain in the face, persistent coughs, swelling of forehead and face areas, nasal obstruction, symptoms related to ears and eye disorders for more than 30 days, no effect of medical treatment during 3 months of treatment of patients, radiographic manifestations in CT scan such as complete turbidity of one or more sinuses, fluid-air level in them and mucosal thickness and obstruction of sinuses, and severe enlargement of nasal tentacles. Patients who were candidate for surgery were hospitalized in the ward one day before the operation. One day later, they were transferred to the operating room for endoscopy, and 3 cc of blood samples was taken from all patients before endoscopy to measure eosinophil and serum immunoglobulin E levels. IgE level of patients was measured by electrochemical luminescence method using Kubas E411 device. The normal range of this immunoglobulin in adults is below100, and more than this number is considered as Hyper-IgE. Samples taken from patients were placed on a slide and fixed. Then, they were stained with Wright's stain. Then, staining process was performed with blue methanol. Patients' samples were washed with water and dried in the open air. Then, 100 cells per slide were assessed by a pathologist, and the number of eosinophils was calculated, and the percentage of these cells was obtained. Nasal eosinophilia is when it is larger than 25% of the cells on the nasal eosinophilia smear. Tissue eosinophil was calculated using the following formula in this way:

(1) $$\begin{array}{c} \text{Tissue Eosinophilia}=\left(\text{Mean eosinophilia count}\right)/\left(\text{Mean total inflammatory}\right)*100. \end{array}$$

After collecting the data, they entered into the SPSS 19 software, and descriptive results were reported by central and dispersion indicators including mean, percentage, frequency, and standard deviation. To normality of the data was first checked by Kolmogorov-Smirnov test (*P* < 0.05), and they were analyzed using Kruskal-Wallis and Mann–Whitney at a significant level of *P* < 0.05.

## 3. Results

**Figure 1:**
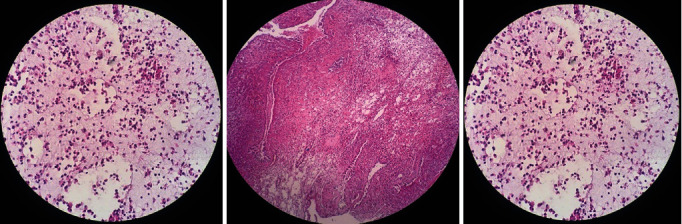
Sections show polypoid lesion covered by respiratory epithelium.

A total of 70 patients had chronic rhinosinusitis, which 43 (61.4%) of them were male and 27 (38.6%) of them were female. Their mean age of them was 39.11 ± 13.72 years. Most of the patients examined in this study were housewives with a frequency of 23 patients (32.9%), and most of the patients lived in urban areas (80%) and were married (80%). Based on the results of this study, the mean serum level of IgE in the studied patients was 232.42 Iu/ml. Also, results of this study indicated that there was no significant difference between patients' gender and mean serum IgE level (*P* < 0.05). The mean blood eosinophil percentage of patients of the present study was 3.81 ± 3.13, and tissue level was 0.35 ± 0.29. Based on the results of table below, most patients (54.3%) in this study had moderate eosinophil level, and 64.3% did not have eosinophilia at the tissue level (Table 1 and Figure 1).

Based on the results of the present study, the mean percentage of eosinophils in blood and tissue samples of patients with chronic sinusitis increased significantly with increasing CT scan score in patients (*P* < 0.05). The results of Pearson correlation coefficient showed that the mean percentage of eosinophils in blood and tissue increased significantly with increasing the CT scan score of patients (*P* < 0.05) (Table 2). The mean percentage of eosinophils in blood and tissue samples of patients with chronic sinusitis did not show a significant change with increasing the patients' clinical symptoms (*P* > 0.05). The results of Pearson correlation coefficient showed that the mean percentage of blood and tissue eosinophils did not increase significantly with increasing clinical score of patients (*P* > 0.05) (Table 3).

## 4. Discussion

In the present study, the mean age of patients of the present study was 39.11 ± 13.72 years, and the majority of patients (61.4%) were male. In a study conducted by Wang et al., the mean age of patients with sinusitis was 40.27 ± 13.47 years, and the majority (72.7%) of them were male [18]. In studies conducted by Kim et al. [19], Tabatabai et al. [20], and Farhadi et al., majority of patients were male with a mean age of 35 to 40 years, which was in line with the results of our study [1]. In our study, the mean serum level of immunoglobulin E in the studied patients was 232.42 ± 360.87 Iu/ml. In a study conducted by Farhadi et al., the mean serum level of IgE in patients with sinusitis was 131.3 ± 140.81 Iu/ml, which mean serum immunoglobulin level was significantly higher than normal level [1]. In a study conducted by Wang et al., the mean serum level of this parameter was 167.97 ± 176.77 Iu/ml, which was higher than the normal range of this immunoglobulin. This result was in line with that of our study [18]. According to results of the present study, there was no significant difference between the mean serum levels of IgE factor and the normal and abnormal levels of serum IgE factor in patients (*P* < 0.05). Results of our study were in line with those of a study conducted by Tabatabai et al., in which no relationship was found between gender and the normal or abnormal serum IgE levels in patients [20]. In our study, the mean percentage of eosinophils in blood samples was 3.81 ± 3.13%, and the mean percentage of tissue eosinophils was 0.35 ± 0.29%. A study conducted by Wang et al. reported that the mean percentage of eosinophils in the blood sample of patients was 3.42 ± 1.87%, and the eosinophil sample of patients' tissue was 0.91 ± 0.8% [18]. Kim et al. also reported that the mean percentage of eosinophils in the studied patients was 2.7% [19]. In a study conducted by Sreeparvathi et al., the mean eosinophil percentage of patients was reported to be 3.6 ± 1.9%, which was consistent with the results of our study [21].

In this study, most of the patients (92.4%) had mild to severe eosinophilia in the blood sample. In a study carried out by Madani et al., 30% of the patients had eosinophilia, which was inconsistent with the results of our study [3]. One of the reasons for this inconsistency in the results might be attributed to geographical location of the patients studied. Also, tissue samples of 36% of patients showed mild eosinophilia. The results of our study are consistent with those of the study conducted by Madani et al., who stated that 42% of patients with rhinosinusitis had mucosal eosinophilia [3]. In a research carried out by Aslan et al., 62.3% of patients had mucosal eosinophilia, which was not in line with the results of our study. One of the reasons for inconsistency in the results might be different definitions of eosinophilia in patients so that serum levels of more than 10 were considered eosinophilia in the mentioned research, while it was considered to be more than 100 in our research [22]. According to the results of present study, the mean eosinophil percentage of patients increased significantly by changing the CT scan score of patients. Also, the results of Pearson correlation coefficient showed that the mean percentage of eosinophils in blood and tissue increased significantly with increasing CT scan score of patients. In a study carried out by Aslan et al., it was reported that the percentage and mean number of eosinophils in patients increased with increasing CT scan score, which is consistent with the results of our study [22].

## 5. Conclusion

No tissue or blood eosinophilia was observed in patients with chronic rhinosinusitis. Also, the mean percentage of blood and tissue eosinophils increased significantly in patients with increasing CT scan score (*P* < 0.05).

## Figures and Tables

**Table 1:** Relative frequency of blood and tissue eosinophil status in patients.

Patient pathology	Eosinophils situation	Frequency	Percent
Blood	None	6	6/8
	Mild	7	0/10
	Moderate	38	3/54
	Severe	19	1/27

Tissue	None	45	3/64
	Mild	25	7/35
	Moderate	0	0
	Severe	0	0

**Table 2:** Comparison of the average percentage of blood and tissue eosinophils in patients according to CT scan score.

Patient pathology	CT scan score status	Average percentage	Kruskal-Wallis statistical test results
Blood	Mild	841/0 ± 920/1	Chi − square = 9.791 Df = 2 *P* = 0.007
	Moderate	408/0 ± 83/2	
	Severe	0/0 ± 0/2	

Tissue	Mild	288/0 ± 362/0	Chi − square = 6.585 Df = 2 *P* = 0.037
	Moderate	148/0 ± 650/0	
	Severe	0 ± 7/0	

**Table 3:** Comparison of the mean percentage of blood and tissue eosinophils in patients according to the condition of clinical symptoms.

Patient pathology	Score status of clinical signs	Average percentage	Kruskal-Wallis statistical test results
Blood	Mild	920/2 ± 738/3	Chi − square = 98.106 Df = 94 *P* = 0.366
	Moderate	78/2 ± 32/3	
	Severe	74/3 ± 3/4	

Tissue	Mild	296/0 ± 340/0	Chi − square = 62.017 Df = 50 *P* = 0.119
	Moderate	293/0 ± 294/0	
	Severe	28/0 ± 43/0	

## Data Availability

The data is available.
